# Spinal cord stimulation as a treatment for refractory neuropathic pain in tethered cord syndrome: a case report

**DOI:** 10.1186/1752-1947-4-74

**Published:** 2010-02-25

**Authors:** Maarten Moens, Ann De Smedt, Jan D'Haese, Steven Droogmans, Cristo Chaskis

**Affiliations:** 1 Department of Neurosurgery, UZ Brussel, Laarbeeklaan, Brussels, 1090, Belgium; 2 Department of Neurology, UZ Brussel, Laarbeeklaan, Brussels, 1090, Belgium; 3 Department of Anesthesiology, UZ Brussel, Laarbeeklaan, Brussels, 1090, Belgium; 4 Department of Cardiology, UZ Brussel, Laarbeeklaan, Brussels, 1090, Belgium; 5 Department of Neurosurgery, CHU de Charleroi, Boulevard Paul Janson, Charleroi, 6000, Belgium

## Abstract

**Introduction:**

The spinal cord is a target for many neurosurgical procedures used to treat chronic severe pain. Neuromodulation and neuroablation are surgical techniques based on well-known specific anatomical structures. However, anatomical and electrophysical changes related to the tethered spinal cord make it more difficult to use these procedures.

**Case presentation:**

We report the case of a 37-year-old Caucasian woman who had several surgical interventions for tethered cord syndrome. These interventions resulted in severe neuropathic pain in her lower back and right leg. This pain was treated by spinal cord stimulation using intra-operative sensory mapping, which allowed the cord's optimal placement in a more caudal position.

**Conclusion:**

The low-voltage and more caudally placed electrodes are specific features of this treatment of tethered cord syndrome.

## Introduction

Tethered cord syndrome (TCS) is a clinical condition caused by prolonged stretching of the lower part of the spinal cord, especially the conus terminalis. It results in the abnormal attachment of the spinal cord to its surrounding tissues. Its clinical manifestations include backache and leg pain (especially with flexion), bowel and bladder dysfunction, lower limb weakness, sensory changes, gait abnormalities, and musculoskeletal deformities of the feet and the spine [1-3]. Primary or congenital causes of TCS can be explained by abnormal secondary neurulation and disorders that are of caudal eminence. On the other hand, acquired causes such as infection, tumor or scars can also lead to tethering [1,3].

The development or progression of symptoms often call for an untethering operation, which involves abnormal anatomy and associated entities like lipomas, myelomeningoceles, lipomyelomeningoceles, dermal sinus and spina bifida occulta [3,4].

Pain is a very common symptom of TCS. The pain worsens with flexion or vigorous physical activity. It affects the lower back, the perineum and/or the legs. Among all the symptoms, however, pain is the one most likely to be improved by surgery, involving a success rate of up to 75% in the adult patients [3].

Unfortunately, complex post-operative pain syndromes are difficult to treat with pharmacological and interventional pain treatments.

One of the more invasive, but effective, treatments for chronic neuropathic pain is neurostimulation. This treatment is based on creating paresthesias due to electrical stimulation in the affected and painful area.

Anatomical changes in the spinal cord, for example, tethered cord syndrome, may influence the exact level and location of electrode implantation.

## Case presentation

**Figure 1:**
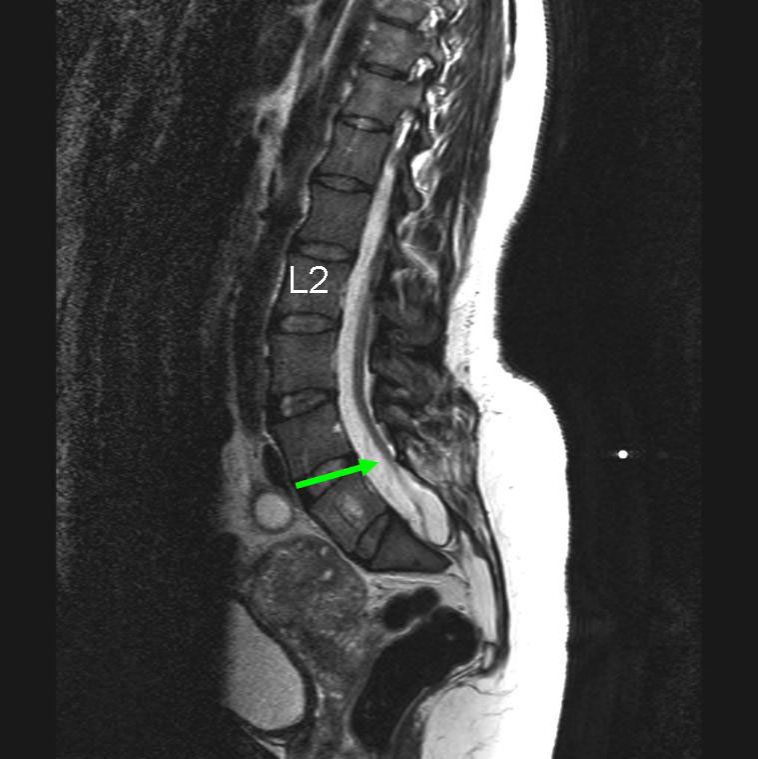
**T2-weighted sagittal magnetic resonance imaging of the lower spinal cord**. Green arrow points to elongated spinal cord.

We report the case of a 37-year-old Caucasian woman (Figure 1) with a history of several surgical interventions for untethering her spinal cord after undergoing a resection of a sacral lipomyelomeningocele at the age of 23. Our patient had a spastic bladder that had recovered partly. Despite these surgeries, however, she has suffered many years of severe chronic pain with heavy burning, dysesthesia and hyperalgesia at her buttocks and her right posterior thigh.

A neurological examination revealed that our patient had no neurological problems besides the sensory deficit at her buttocks and leg and her hyper-reflexive neurogenic bladder problem. She scored 7 on the DN4 questionnaire for indicating neuropathic pain [5]. Her treatment with carbamazepine, pregabalin, gabapentin and fentanyl was not effective. Her other treatment with physiotherapy and psychological guidance neither changed nor reduce her pain, and only high-frequency transcutaneous electrical nerve stimulation (TENS) partly diminished her pain.

**Figure 2:**
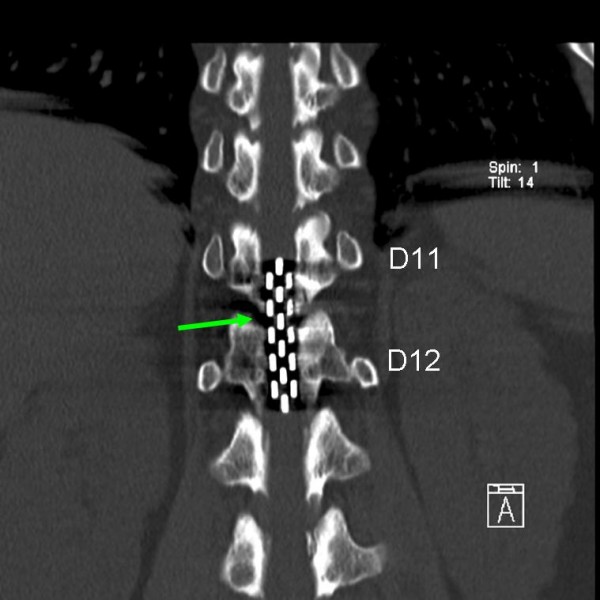
**Coronal view of computed tomography scan of the lower dorsal spine**. Green arrow points to Specify 565 electrode at levels D11 and D12.

Subsequently, a spinal cord stimulator (SCS) was surgically placed when our patient was under epidural anesthesia. An epidural catheter was inserted at levels L2 to L3. Our patient was injected with a loading dose of 0.5% ropivacaine with 0.5 μg/ml sufenta. She was also administered with top-up doses of 4 ml 0.5% ropivacaine to reach a segmental sensory block. We also performed a mid-line flavectomy at levels T10 to T11 and orthodromically inserted a Specify 565 electrode (Medtronic Inc., Minneapolis, Minnesota) at levels T9 to T10 and T11-T12 (retrograde). Using intra-operative stimulation, we performed a mapping of our patient's sensory responses to epidural stimulation, all the while searching for the best level of stimulation. At the higher levels (T9 to T10) we noted paresthesias to her loins, abdominal wall and anterior part of the upper leg but not to her buttock or posterior thigh. Paresthesias were only achieved by stimulation at level T12. Therefore, the Specify 565 electrode was centered at level T12 for definitive implantation (Figure 2).

Following the operation, the intensity of the pain she felt improved from 9 pre-operative to 2 post-operative on the visual analogue scale (VAS). She was able to reduce her painkillers substantially and required a daily dose of just 500 mg of paracetamol. Her definitive SCS parameters were from 0.1 to 0.2 V for amplitude, 60 Hz for frequency, and 240 μsec for pulse width.

## Discussion

The effectiveness of SCS in patients with chronic intractable neuropathic pain is well-known and comprehensively described [6].

According to the authors' expertise and preference, the placement of surgical plate electrodes is the choice of implantation. This placement offers a broader stimulation pattern, lower stimulation requirements, better long-term effectiveness, and lower migration rate. Such are the technical advantages of plate electrodes as compared with percutaneously implanted electrodes [7-9].

Intra-operative stimulation is the cornerstone of any successful procedure. Patients should be able to perceive stimulation in areas where they feel pain. Patients, therefore, must be awake, feel comfortable without any pain, and fully cooperative to report this to the implant team during the placement of electrodes [10,11]. Epidural anesthesia is the technique of choice when using minimally invasive flavectomy, because it provides better hemodynamic stability. Compared to subarachnoid anesthesia, epidural anesthesia also promotes the possibility of extending intra-operative anesthesia through the epidural catheter without any meningeal puncture [12]. With this type of anesthesia patients can feel paresthesias during intra-operative stimulation because local epidural anesthetic acts mostly at the nerve roots level and do not completely block the spinal cord [12].

Technically, the coverage of plate electrodes is limited to 5 levels (2 to 3 levels in orthodromical direction and 2 levels in retrograde sense) depending on the type of electrode. Therefore, guidelines to direct the current flow more precisely and to stimulate the desired body areas are necessary. Barolat *et al*. (1991) published data on spine levels of cathode in connection with specific body areas. They concluded that areas difficult to stimulate are the neck, the lower back, and the perineum. For stimulation of the buttock, electrodes can be placed in a range of T10 to L1 with corresponding stimulation pattern at levels T11 to T12. It must be activated first on the posterior leg fibers, then on the posterior thigh, and lastly on the buttock [13].

In our daily practice, we usually place the electrodes at levels T9 to T10 in order to stimulate our patient's whole leg and lower back. But in this case, because of the stretching of the lower part of our patient's spinal cord, we performed a sensory mapping of the spinal cord. As expected, our patient felt the paresthesias in the examined regions when they were stimulated one level below the usual level but in the normal range as described by Barolat *et al*. (1991).

We may hypothesize that the anatomical stretching in TCS is more extended than the electrophysiological and functional tensions. *In vitro*, experiments showed that maximal cord elongation occurs at the lumbosacral region, some elongation at the thoracic area, and minimal to none at all at the cervical region [14]. Electrophysiological testing in more patients should be undertaken in order to generalize this hypothesis.

Another point that was observable in our patient's case is the low voltage used to obtain excellent pain relief. This can also be explained by the anatomy of the spinal cord in TCS. Due to the attachments or scars, the spinal cord is now placed at a more posterior place and thus in closer contact with the dura mater. It is proven that the voltage needed for the recruitment of nerve fibers, and thus the perception threshold of paresthesia, is related with the distance between the electrode and the spinal cord [15].

The low voltage needed to relieve pain lowers energy consumption and favors a longer battery life. In the end it benefits the cost effectiveness of SCS in patients with TCS.

The implantation of a spinal cord stimulator in a patient with TCS as an effective treatment for refractory chronic neuropathic pain has not been described previously. Despite the anatomical abnormality of the spinal cord in TCS, neuromodulation is an effective therapeutic option to achieve pain relief.

Depending on the severity of the tethered cord, the electrode must be implanted more caudally than in cases involving normal spinal cord. In our opinion, this and its low voltage requirement are the two main elements for the successful treatment of TCS using neuromodulation.

## Conclusion

We reported for the first time a case of sensory mapping for SCS in the treatment of neuropathic pain in TCS. We successfully implanted the epidural electrode in a more caudal position than usual, while using a lower voltage to obtain the best response.

## Abbreviations

SCS: spinal cord stimulation; TCS: tethered cord syndrome; TENS: transcutaneous electrical nerve stimulation; VAS: visual analogue scale.

## Consent

Written informed consent was obtained from our patient for publication of this case report and any accompanying images. A copy of the written consent is available for review by the Editor-in-Chief of this journal.

## Competing interests

The authors declare that they have no competing interests.

## Authors' contributions

MM performed the implantation, analyzed data of our patient and drafted the manuscript. ADS examined our patient and was the major contributor in writing the manuscript. JDH performed the intra-operative testing and was a major contributor in writing the manuscript. SD was involved in the critical revision of the manuscript for important intellectual content. CC involved in supervision. All authors read and approved the final manuscript.
